# Dupilumab-Associated Head and Neck Dermatitis With Ocular Involvement in a Ten-Year-Old With Atopic Dermatitis: A Case Report and Review of the Literature

**DOI:** 10.7759/cureus.27170

**Published:** 2022-07-23

**Authors:** Arthur M Samia, Lyda Cuervo-Pardo, Marjorie E Montanez-Wiscovich, Vanessa Y Cavero-Chavez

**Affiliations:** 1 Dermatology, University of Florida, Gainesville, USA; 2 Allergy and Immunology, University of Florida, Gainesville, USA; 3 Dermatology, University of Florida College of Medicine, Gainesville, USA; 4 Pediatrics, University of Florida, Gainesville, USA

**Keywords:** monoclonal antibodies, dupilumab, conjunctivitis, child, atopic dermatitis

## Abstract

Facial and neck erythema secondary to dupilumab use is a side effect not reported in clinical trials; however, it has been reported aftermarket initially in adults and most recently in adolescents. We report the youngest known case of head and neck dermatitis (HND) secondary to *Malassezia furfur* accompanied by ocular involvement. Treatment with oral fluconazole 150 mg weekly was initiated with subsequent cutaneous improvement. Additionally, his conjunctivitis improved with fluorometholone 0.1% eye drops. As dupilumab becomes more accessible to children, understanding the pathophysiology of HND, characterizing the clinical course, and developing diagnostic and treatment guidelines for this age group will be imperative.

## Introduction

Atopic dermatitis (AD) is a chronic, inflammatory skin disease characterized by pruritus and skin erythema, dryness, oozing, crusting, and eventually lichenification [1]. It is more frequent in children than adults, with approximately 15-20% and 1-3% affected, respectively [2]. The American Academy of Dermatology recommends treating AD with non-pharmacologic interventions (e.g., moisturizers, appropriate bathing practices), topical pharmacological preparations (e.g., corticosteroids, calcineurin inhibitors, phosphodiesterase-4 inhibitors), and phototherapy if necessary and available [3-5]. Historically, systemic therapies (e.g., cyclosporine, azathioprine, methotrexate, mycophenolate mofetil) have been reserved for moderate to severe pediatric AD refractory to standard therapies [6,7]. However, these medications were used off-label and required laboratory monitoring, had unfavorable side effect profiles, and resulted in high recurrence rates, as in the case of oral prednisone. Dupilumab was the first systemic agent available for pediatric patients and was approved in 2019 to treat children over 12 years old with moderate to severe AD [3-5]. Its use was extended to children over six years old in 2020 [8], and more recently in 2022, to those six months and older.

Dupilumab is a monoclonal antibody that binds the interleukin (IL) 4 receptor’s alpha subunit to inhibit IL-4 and IL-13 signaling, which reduces T helper cell (Th) 2-mediated inflammation [9]. Dupilumab has a favorable side effect profile and is a long-term therapeutic option for AD [10]. Several studies have verified dupilumab’s safety and efficacy for treating AD [11]. Clinical trials reported primarily mild to moderate, reversible adverse effects, including injection site reactions, conjunctivitis, eosinophilia, and new-onset psoriasiform dermatitis [11]. Facial and neck erythema, also known as dupilumab facial redness, is a side effect not reported in clinical trials; however, it has been reported aftermarket in 4-10% of adults after a median of 65 days from starting dupilumab and more recently in children and adolescents [12-14]. While the pathogenesis of dupilumab facial redness is unknown, the proposed etiologies for facial dermatitis include the unmasking of psoriasis, rosacea, allergic contact dermatitis (ACD), and head and neck dermatitis (HND) associated with *Malassezia furfur (M. furfur)* [8]. We report the youngest known case of dupilumab-associated HND accompanied by ocular involvement [8,12].

## Case presentation

**Figure 1:**
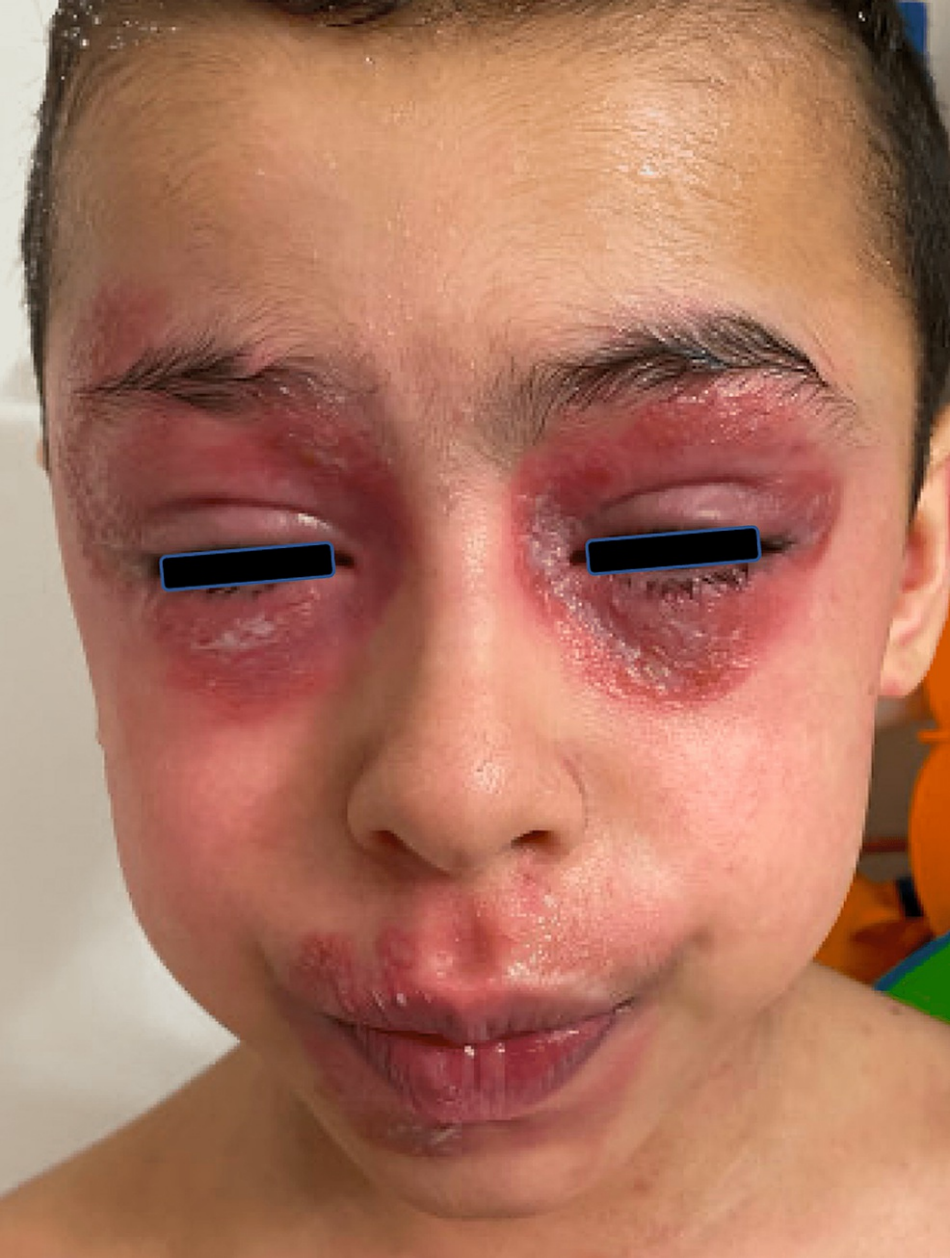
Pre treatment full-face involvement

**Figure 2:**
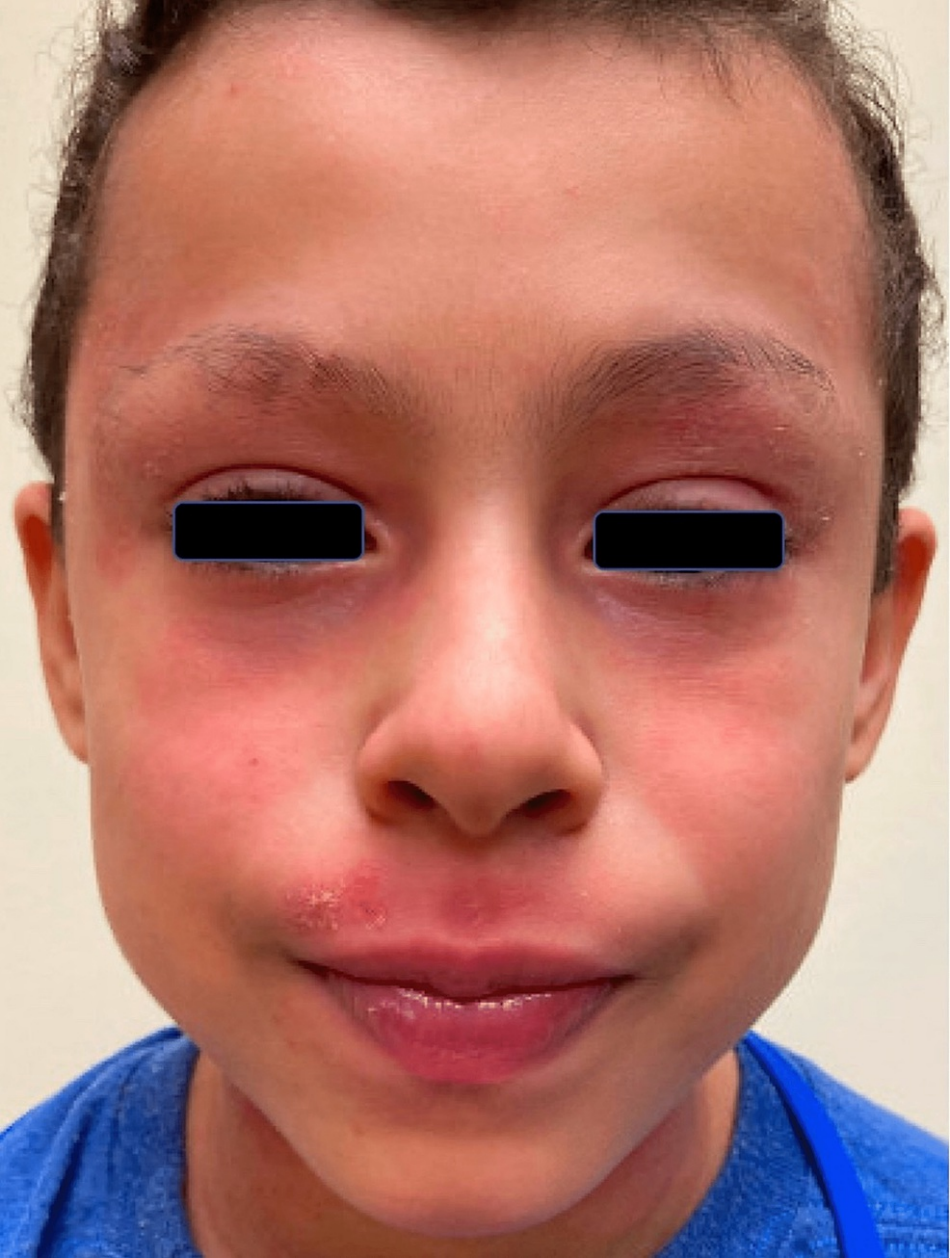
Clinical improvement post treatment

A ten-year-old boy with a history of severe AD ongoing since infancy presented to the allergy clinic for disease management. His AD had been refractory to several potent topical corticosteroids, topical calcineurin inhibitors, and phototherapy. He never required hospitalization for his AD; however, he experienced several episodes of impetiginization, which resolved with topical mupirocin 2% ointment. Due to his recalcitrant disease, he was started on dupilumab 200mg every two weeks with a significant early improvement of AD of the trunk and extremities. After six weeks of dupilumab, he developed marked erythema on the head and neck that progressed to form erythematous plaques with mild scale and crust on superior and inferior eyelids with associated perioral skin dermatitis and yellow-tinged ocular secretions (Figure 1). There were no other areas of new regional dermatitis, urticaria, or mucosal involvement. Following a review of the literature, treatment with oral fluconazole 150mg weekly for eight weeks and clotrimazole 1% cream applied to the face was initiated, which improved his dermatitis (Figure 2). Conjunctivitis improved after treatment with fluorometholone 0.1% eye drops. Dupilumab was discontinued at the onset of lesions for five weeks and restarted once HND improved. While off dupilumab, AD was exacerbated on both arms and the right popliteal fossa. After restarting dupilumab, his AD became well controlled with the development of only one episode of mild facial erythema managed with clotrimazole 1% cream as needed. To further elucidate his HND's etiology and evaluate the potential contribution of ACD, the patient underwent patch testing to the pediatric patch test series [15]. He had weakly positive reactions to decyl glucoside and nickel sulfate hexahydrate, doubtful reactions to amidoamine and carmine, and an irritant reaction to dimethylaminopropylamine. However, the patient was tested on dupilumab, and some reactions may have been fully or partially suppressed [16].

## Discussion

Three studies have evaluated dupilumab-associated HND in children and adolescents, suggesting HND development in 5.4-29% of cases [8,12,14]. Muzumdar et al. published the first dedicated study evaluating dupilumab-associated HND in children [12]. They evaluated 24 children receiving dupilumab injections for AD, of which seven (29%) had new-onset or worsening HND after starting dupilumab [12]. Additionally, an off-label use trial evaluating dupilumab for AD between ages three and 18 reported HND in 6/111 (5.4%) of patients [14]. Muzumdar et al. observed HND had a higher incidence with increasing age - 6/17 (35%) post-pubertal while only 1/7 (14%) pre-pubertal patients presented with HND [12]. HND’s onset occurred six or fewer months after starting dupilumab in 2/7 (29%) cases and after greater than six months in the remaining 5/7 (71%) cases [12]. Our patient’s onset of HND occurred six weeks after starting dupilumab, which is consistent with this data.

Bax et al. described HND in five cases of patients between the ages of 12 and 19 [8]. The HND case ages, genders, and presence of concomitant ocular involvement in Bax et al. and Muzumdar et al.’s studies are summarized in Table 1 [8,12]. Overall, HND appeared more prevalent in females and as children reached adolescence [8,12]. Additionally, ocular involvement did not appear to be typical [8,12]. Based on this data, our case is relatively unique given the patient’s age and pre-pubertal status, gender, and ocular involvement.

**Table 1:** Summary of HND case ages, genders, and presence of concomitant ocular involvement in Bax et al. and Muzumdar et al.’s studies

	Bax et al. [8]	Muzumdar et al. [12]
Age		
≤10 years	0/5 (0%)	1/7 (14%)
11-15 years	3/5 (60%)	3/7 (43%)
16-19 years	2/5 (40%)	3/7 (43%)
Gender		
Male	0/5 (0%)	3/7 (43%)
Female	5/5 (100%)	4/7 (57%)
Ocular involvement	1/5 (20%)	1/7 (14%)

Bax et al. also described treatment outcomes in their case series [8]. All five patients were treated with fluconazole 150 mg weekly for two weeks in two cases and four weeks in three cases [8]. Clotrimazole 1% cream was used in one case, and triamcinolone 0.025% cream was used in another [8]. There was a recurrence of symptoms in two cases; however, only one patient was re-treated with fluconazole 150mg weekly for four additional weeks, which led to a successful resolution of symptoms [8]. Based on this review, it was decided to extend the duration of fluconazole therapy from four to eight weeks in our case. Dupilumab was noted to be discontinued in two cases, then restarted following a resolution of symptoms in one case [8]. In three cases, there was worsening of symptoms with fluconazole cessation and improvement after it was restarted. Muzumdar et al. reported that all their pediatric HND patients were empirically treated with ketoconazole 2% cream; however, outcomes were not discussed [8]. Another case study reported resolution of symptoms in two adult patients with HND treated with oral itraconazole [17]. Bax et al.’s findings and this second case series support the possible *M. furfur*-associated component of HND, given the seborrheic distribution of HND in these cases and positive response to oral antifungals. Although, improvement in these patients may be attributable to fluconazole’s suggested anti-inflammatory properties rather than anti-mycotic properties [18].

The proposed pathophysiologic mechanism behind the *M. furfur*-induced HND theory is driven by dupilumab’s Th2 blockade via the IL-4 alpha receptor skewing toward a Th17-mediated inflammatory response, thus potentiating *M. furfur*-related hypersensitivity and inflammation [12,19]. This theory has been further supported by the identification of a statistically significant increase in baseline *Malassezia*-specific serum IgE in subjects who developed dupilumab-associated HND compared to subjects who did not [20]. The identification of *Malassezia*-specific serum IgE over 0.35 kU/mL has been associated with a sensitivity of 94.1% and an 87.9% specificity for the diagnosis of dupilumab-associated HND and may be considered as a biomarker to predict dupilumab-associated HND prior to initiating therapy [20]. Our case correlates with this theory, given the patient’s HND in a typical seborrheic distribution and successful resolution of symptoms after fluconazole therapy. However, the patient’s pre-pubertal status is atypical given the association of HND with seborrheic dermatitis and seborrheic dermatitis’s typical pubertal or post-pubertal onset [21].

Other proposed etiologies of dupilumab-induced facial dermatitis include unmasking ACD, psoriasis, or rosacea [8]. Our patient completed patch testing, which showed reactions to decyl glucoside, dimethylaminopropylamine, and amidoamine. These were deemed to be of possible relevance given their role as surfactants or impurities in hair shampoos, which correlate with the predominant face and neck distribution of the patient’s eruption. It has been theorized that varying degrees of IL-4 and IL-13 inhibition may reveal suppressed ACD to an allergen [22]. Alternatively, the shift to primarily Th1 and Th17 immune response pathways with dupilumab may unmask rosacea by promoting Demodex proliferation [23]. These theories are important to consider as HND’s causal relationship with *M. furfur* has not been adequately established. Further emphasizing this point, only one of Bax et al.’s patients had a skin biopsy from an area of active HND, and the findings were not supportive of an apparent *M. furfur* burden [8].

Conjunctivitis is the second most common side effect reported with dupilumab injections after injection site reactions, and ocular involvement occurring concurrently with HND appears relatively common as well [8,11,12]. During clinical trials, conjunctivitis was reported in 9-11% of patients receiving dupilumab injections every other week [9]. Out of the 13 HND cases reported by Bax et al., Muzumdar et al., and in our case, three (23%) presented with ocular involvement [8,12]. Bax et al.’s 14-year-old patient was followed by ophthalmology for her ocular involvement [8]. A relationship between patient age and concomitant ocular involvement has not been established. Given the relatively low population of pediatric patients with HND, it is not possible at this time to make any statistical associations between ocular involvement in HND and pediatric status.

## Conclusions

This is the youngest known case of dupilumab-associated HND and conjunctivitis. Resolution of symptoms with fluconazole suggests *Malassezia furfur* may play an important role in HND pathophysiology, although anti-inflammatory properties of antifungals and other potential etiologies should still be considered. Overall, oral antifungals appear effective for treating pediatric patients with dupilumab-associated HND. Additionally, topical antifungal therapy may have a role in maintenance therapy. When ocular involvement is present, fluorometholone 0.1% eye drops and follow-up with ophthalmology appear to be effective management tools, as was true in our case. Further investigation may be warranted to elucidate a possible association between younger patient age and HND with ocular involvement. As dupilumab becomes more accessible to younger children, understanding the pathophysiology of HND, characterizing its clinical course, and developing workup and treatment guidelines for this age group will be imperative.
